# ALDH1a3 Protects Against Iron Overload−Induced Oxidative Stress and Mitochondrial Impairment in Renal Tubular Epithelial Cells

**DOI:** 10.3390/antiox15050577

**Published:** 2026-05-02

**Authors:** Tingting Wei, Zongliang Xiong, Tianci Wang, Chao Huang, Qihui Luo, Riyi Shi, Lanlan Jia, Wentao Liu, Donghui Yang, Zhengli Chen

**Affiliations:** 1 Laboratory of Experimental Animal Disease Model, College of Veterinary Medicine, Sichuan Agricultural University, Chengdu 611130, China; 2 Key Laboratory of Animal Disease and Human Health of Sichuan Province, College of Veterinary Medicine, Chengdu 611130, China; 3 Center for Paralysis Research, Department of Basic Medical Sciences, College of Veterinary Medicine, Purdue University, West Lafayette, IN 47907, USA

**Keywords:** iron overload, oxidative stress, ALDH1a3, mitochondrial dysfunction, dimercaprol

## Abstract

Iron overload significantly contributes to chronic kidney disease progression by triggering oxidative stress and mitochondrial impairment via the Fenton reaction. This study investigates the protective role of aldehyde dehydrogenase 1a3 (ALDH1a3), an enzyme that detoxifies reactive aldehydes, in renal iron overload. C57BL/6N mice were fed a 2.25% ferric citrate diet for 24 weeks to establish a chronic model, followed by treatment with the chelator Dimercaprol (DP). In vitro, TCMK−1 cells were subjected to iron intervention with ALDH1a3 overexpression or inhibition. Chronic iron overload induced significant renal iron deposition, lipid peroxidation (elevated MDA, depleted GSH), and mitochondrial structural damage. ALDH1a3 was endogenously upregulated in renal tubular epithelial cells under iron stress. Overexpressing ALDH1a3 significantly enhanced cell viability, suppressed reactive oxygen species and MDA levels, and preserved mitochondrial membrane potential, whereas its inhibition exacerbated cellular damage. Furthermore, DP treatment reduced iron deposition and was associated with increased ALDH1a3 expression. In conclusion, ALDH1a3 acts as a critical endogenous protective factor against iron−induced nephrotoxicity by mitigating oxidative damage and maintaining mitochondrial stability. These findings indicate that ALDH1a3 is a promising potential therapeutic target for the treatment of iron overload−related kidney diseases.

## 1. Introduction

Iron is an indispensable element for the organism, maintaining homeostasis through tightly regulated absorption, circulation, storage, and excretion processes [1]. However, when iron supply exceeds demand, homeostasis is disrupted, leading to a state known as iron overload, which poses a continuous threat to tissues and cells [2,3]. Chronic iron overload can result from long−term high−iron diets, iron supplementation, hereditary hemochromatosis, or frequent blood transfusions [4,5,6]. Under iron overload conditions, free iron ions generate hydroxyl radicals—highly reactive oxygen species (ROS)—via Fenton and Haber–Weiss reactions. These radicals indiscriminately attack biological macromolecules such as proteins, lipids, and DNA, leading to structural damage and functional loss. Specifically, the attack on polyunsaturated fatty acids in cell membranes initiates a cascade of lipid peroxidation, destroying membrane structure and producing various toxic aldehyde byproducts (e.g., malondialdehyde, MDA), which further damage proteins and DNA [7,8,9,10].

Abnormal iron metabolism and oxidative stress−mediated dysfunction are involved in the progression of various chronic kidney diseases (CKD) [11,12,13]. Ferric citrate (FC), an FDA−approved oral phosphate binder, is commonly used to treat iron−deficiency anemia in non−dialysis CKD patients and hyperphosphatemia in end−stage renal disease (ESRD) patients [14,15,16]. However, the potential risk of organ iron overload associated with FC requires attention. Clinical observations have reported iron overload in peritoneal dialysis patients treating hyperphosphatemia with FC [17]. Furthermore, FC treatment has been shown to increase ferritin concentrations more significantly than ferrous sulfate in patients with moderate to severe CKD [15]. Excess iron often triggers cell injury and death through oxidative stress, exacerbating renal damage [18]. Due to their specific reabsorption functions and high metabolic demand, renal tubules are particularly susceptible to high−iron environments and iron−induced oxidative injury [19,20]. Studies indicate that iron sucrose significantly increases intracellular iron content and hydroxyl radical content in HK−2 cells while upregulating fibrosis markers [21]. Additionally, as renal tubules have a higher mitochondrial density than other nephron segments, the interaction between superoxide anions from the mitochondrial respiratory chain and free iron amplifies oxidative stress signals, forming a vicious cycle that eventually overwhelms the antioxidant defense system [22].

Aldehyde Dehydrogenase 1 Family Member a3 (ALDH1a3) has recently been identified as a key player in cellular defense [23,24,25]. Beyond its roles in stem cell differentiation and retinoic acid synthesis [26,27,28], ALDH1a3 detoxifies reactive aldehydes produced as byproducts of lipid peroxidation, thereby mitigating oxidative stress [29,30,31,32,33]. This function appears particularly relevant in the context of iron overload. The generation of toxic aldehydes during iron−induced lipid peroxidation and mitochondrial dysfunction may trigger ALDH1a3 expression or activity as an adaptive response [31]. However, direct research on the role of ALDH1a3 in renal iron overload remains limited. Based on existing literature, we hypothesize that ALDH1a3 exerts protective effects in iron−overloaded renal cells through multiple pathways: clearing toxic aldehydes to preserve cell morphology and function [23]; regulating mitochondrial function to influence ROS production and ferroptosis sensitivity [34]; and potentially participating in retinoic acid metabolism to modulate stress responses [35].

Dimercaprol (DP) is a dithiol compound whose main function is to form stable five−membered rings with certain heavy metals through its thiol groups, thereby neutralizing toxicity and promoting metal excretion. DP has been used to treat poisoning by arsenic, mercury, gold, lead, antimony, and other toxic metals, as well as Wilson’s disease caused by copper ions [36]. Acrolein, as a known product of free radical−induced lipid peroxidation (LPO), also serves as a key factor in sustaining oxidative stress [37]. Regarding the detoxifying effects of acrolein, DP has been found to significantly protect the neuronal cells PC−12 from acrolein−mediated cell death in a dose−dependent manner [38]. Iron overload is precisely a form of cellular oxidative damage mediated by iron ions. Therefore, these findings suggest that DP may hold significant potential for treating iron overload; however, its effective and safe dosage for renal use, as well as its specific mechanism of action, remains unknown.

In this study, we established a chronic iron overload mouse model using ferric citrate to investigate the specific characterization of renal injury. We conducted functional studies on ALDH1a3 to determine its protective role against iron−induced oxidative stress and mitochondrial injury. Furthermore, we compared the protective effects of ALDH1a3 with the metal chelator Dimercaprol (DP). Our study reveals that ALDH1a3 plays a positive role in resisting iron−induced oxidative stress and mitochondrial injury in renal tubular epithelial cells, identifying it as a novel key protective factor. We propose that ALDH1a3, through direct antioxidant mechanisms, may serve as a complementary or alternative target to DP for the intervention of iron overload−related kidney diseases.

## 2. Materials and Methods

### 2.1. Animal Administration

Ninety−five 8−week−old male C57BL/6N mice (Vital River Laboratories, Chengdu, China) were housed in an SPF environment (18–22 °C, 50–60% humidity, 12 h light/dark cycle). After one week of acclimatization, mice were randomly assigned to a Control group (Ctrl, *n*= 25) fed a standard diet, and a Model group (FC, *n*= 70) fed a diet supplemented with 2.25% ferric citrate. After 24 weeks, 10 mice from each group were sacrificed for analysis. The remaining mice (15 Ctrl, 60 FC) were switched to a normal diet. The FC mice were subdivided into DP−L (4 mg/kg), DP−M (8 mg/kg), and DP−H (16 mg/kg) groups and treated daily via intraperitoneal injection (3:00–4:00 PM) with Dimercaprol dissolved in PBS. The Ctrl group received PBS vehicle. Treatment continued for 4 weeks.

### 2.2. Blood Biochemistry

Serum was separated by centrifugation (3000 rpm, 15 min, 2–8 °C) after clotting. Urea and Creatinine (Crea) were measured using an automatic biochemical analyzer (Rayto, Shenzhen, China). GSH was measured using a microplate reader kit (A006−2, Nanjing Jiancheng, Nanjing, China).

### 2.3. Tissue Collection

Mice were anesthetized with Zoletil. Kidneys were weighed, and samples were either snap−frozen in liquid nitrogen for molecular analysis or perfused with PBS followed by 4% PFA for histological fixation.

### 2.4. Histopathology and Staining

Paraffin−embedded kidney sections (5 μm) underwent Hematoxylin and Eosin (H&E) staining for morphological assessment. Iron deposition was visualized using Prussian Blue staining (G1429, Solarbio, Beijing, China). Stained sections were imaged and analyzed.

### 2.5. RNA Isolation and Quantitative Real−Time PCR

Total RNA was isolated from Kidney using the Animal Total RNA Isolation Kit (RE−03014, FOREGENE, Chengdu, China). RNA quality was assessed by A_260_/A_280_ ratio. One μg of total RNA was reverse−transcribed into cDNA. qRT−PCR was performed using Real Time PCR EasyTM−SYBR Green I (QP−01011, FOREGENE). Primers for Havcr−1 were: F:5′−TCAGGGTCTCCTTCACAGCA−3′ and R:5′−GCTCACAAGGAGCAGTAGCA−3′. β−actin served as the endogenous control. Relative expression was calculated using the 2^−∆∆Ct^ method.

### 2.6. Transcriptomic Analysis

Transcriptomic sequencing and bioinformatics analysis were performed by Shanghai Applied Protein Technology Co., Ltd. (Shanghai, China). Following quality control of total RNA extracted from tissue samples, qualified samples were processed for library construction. Eukaryotic mRNA was enriched using Oligo (dT) magnetic beads and fragmented randomly using fragmentation buffer. First−strand cDNA was synthesized using random hexamer primers, followed by second−strand cDNA synthesis. The synthesized cDNA was purified, end−repaired, A−tailed, and ligated with sequencing adapters. The fragments were then selected for size and enriched via PCR to generate the final cDNA library. After validating insert size and concentration, the libraries were pooled and sequenced on the Illumina platform.

### 2.7. Western Blot

Kidney tissues were lysed, and protein concentrations were normalized. Samples were separated by SDS−PAGE, transferred to PVDF membranes, blocked, and incubated with primary antibodies overnight at 4 °C, followed by secondary antibodies. Bands were visualized using a gel imaging system and analyzed with Image J version 2.14. β−actin served as the loading control.

### 2.8. Immunofluorescence

Paraffin sections were deparaffinized, subjected to antigen retrieval (citrate buffer), and blocked. Sections were incubated with primary antibodies (1:1000) overnight at 4 °C, followed by fluorescent secondary antibodies (1:1000). Nuclei were stained with DAPI.

### 2.9. Antibodies

Rabbit anti−ALDH1a3 was sourced from (25167−1, Proteintech, Wuhan, China) and (A15230, ABclonal, Wuhan, China); rabbit anti−FTL (A11241, ABclonal); rabbit anti−Transferrin (17435−1−AP, Proteintech); anti−β−actin (Ac026, ABclonal); anti−GAPDH (A19056, ABclonal); anti−ACSL4 (T510198, Abmart, Shanghai, China); anti−GPX4 (ABclonal, A25009); anti−4HNE (ab48506, Abcam, Cambridge, UK).

### 2.10. Biochemical Assays (SOD, MDA, CAT, Total Antioxidant Capacity)

Kidney tissues or cells were homogenized in PBS. Supernatants were collected after centrifugation. Total SOD activity (S0101S, Beyotime, Shanghai, China), MDA levels (S0131S, Beyotime), CAT activity (S0051, Beyotime) and total antioxidant capacity (S0121, Beyotime) were measured according to manufacturer protocols using a microplate reader. It should be noted that the SOD assay we conducted primarily measures total SOD activity.

### 2.11. Determination of GSH and GSSG Levels

TCMK−1 cells were PBS−washed, lysed in protein removal reagent M, and centrifuged. For GSSG assay, samples underwent GSH scavenging at 25 °C for 60 min. Standard curves were prepared with GSSG dilutions. In 96−well plates, samples/standards mixed with protein removal reagent M and total glutathione working solution, incubated at 25 °C for 5 min, then NADPH added. Absorbance at 412 nm measured every 5 min for 25 min. GSH = total glutathione—2 × GSSG.

### 2.12. Cell Culture and Treatment

TCMK−1 cells (SNL−602, Sunncell, Wuhan, China) were cultured in MEM with 10% FBS and 1% Pen/Strep. Cells were treated with Ferric Ammonium Citrate (FAC) at various concentrations for 12 h. Transfections were performed using LipoMaxTM. For inhibition, cells were pretreated with ALDH1A3−IN−3 (HY−W017186, MCE, Shanghai, China) for 2 h.

### 2.13. Cell Viability, ROS, and Mitochondrial Membrane Potential

Viability was assessed using the CCK−8 assay (C0038, Beyotime). ROS levels were visualized using DCFH−DA probes. Mitochondrial membrane potential was evaluated using JC−1 staining (C2003S, Beyotime). Images were captured using an inverted fluorescence microscope.

### 2.14. Statistical Analysis

Data were analyzed using GraphPad Prism 10 and expressed as mean ± SD. Group comparisons were performed using one−way ANOVA or *t*−tests. *p* < 0.05 was considered statistically significant.

## 3. Results

### 3.1. Chronic Iron Overload Induces Injury and Oxidative Stress in Mouse Renal Tubular Epithelial Cells

**Figure 1:**
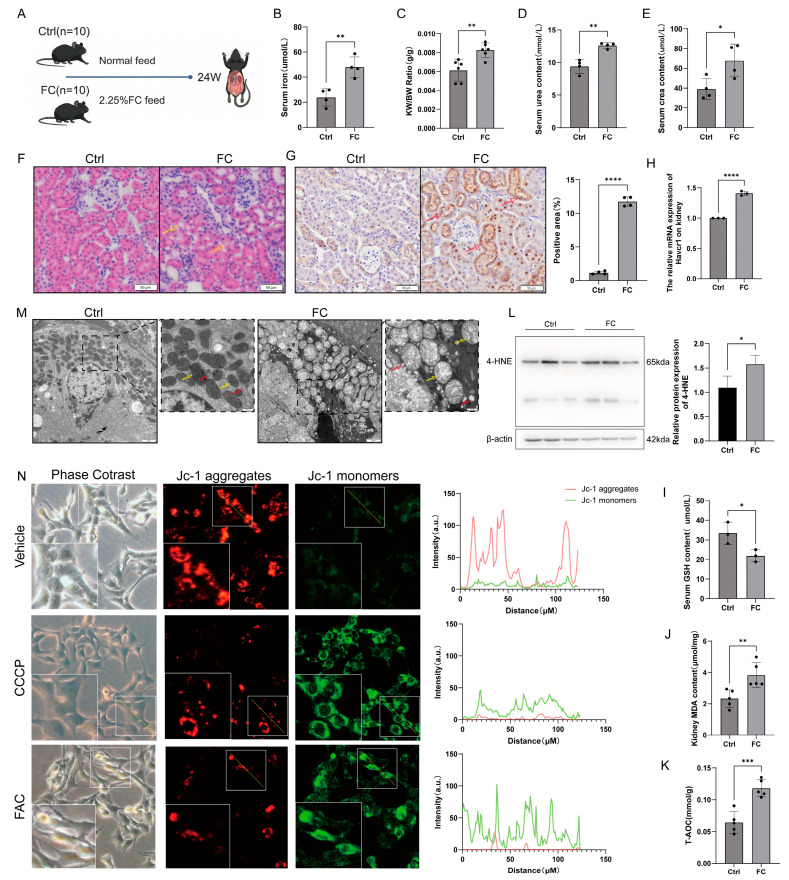
Iron overload induces renal injury and oxidative stress in mice. (**A**) Experimental timeline: C57BL/6N mice fed normal (Ctrl) or 2.25% ferric citrate (FC) diet for 24 weeks. (**B**) Serum iron levels. (**C**) Kidney/body weight ratio. (**D**) Serum Urea levels. (**E**) Serum Crea levels. (**F**) H&E staining of kidney sections; the yellow arrows indicate cytoplasmic vacuolation. (**G**) Prussian Blue staining and quantification of iron deposition; the red arrow indicates iron deposition. (**H**) Havcr1 mRNA levels. (**I**) Serum GSH. (**J**) Renal MDA. (**K**) Renal total antioxidant capacity. (**L**) Western blot and quantification of 4−HNE in the kidneys of iron−overloaded mice. (**M**) Transmission electron microscopy of mitochondrial morphology in tubular cells; the yellow arrows indicate mitochondria, and red arrows indicate the rough endoplasmic reticulum. (**N**) Mitochondrial membrane potential (JC−1) in TCMK−1 cells treated with 200 μM FAC. The red fluorescence indicates a higher mitochondrial membrane potential, while the green fluorescence indicates a lower mitochondrial membrane potential. The white box displays the enlarged view. All data are shown as mean ± standard deviation (SD). Statistical comparisons were performed using one−way ANOVA or t−tests. * *p* < 0.05, ** *p* < 0.01, *** *p* < 0.001, **** *p* < 0.0001.

To establish a chronic iron overload model, C57BL/6N male mice were fed either a normal diet (Ctrl) or a diet containing 2.25% ferric citrate (FC) for 24 weeks (Figure 1A). After 24 weeks, serum iron levels were elevated in the FC group, indicating systemic iron deposition (Figure 1B). Comparison of organ indices and renal function revealed a significant increase in kidney weight in the FC group (Figure 1C), alongside elevated serum Urea and Crea levels (Figure 1D,E), indicating severe renal dysfunction. Histological analysis via HE staining and Prussian Blue staining revealed obvious cytoplasmic vacuolation in the renal tubules of the FC group (Figure 1F), with iron deposition primarily localized to the tubular epithelial cells (Figure 1G). Expression of the tubular injury marker Havcr1 was also significantly increased (Figure 1H). These results confirm that iron−induced damage significantly impacts renal tubular epithelial cells.

Iron deposition is typically accompanied by cellular oxidative stress. In the FC group, systemic iron accumulation led to the consumption of serum glutathione (GSH) (Figure 1I), a significant increase in renal MDA (a lipid peroxidation product) (Figure 1J), and an unexpected increase in total antioxidant capacity (Figure 1K), suggesting the activation of compensatory antioxidant programs. In the kidneys of iron−overloaded mice, we also observed increased expression of 4−HNE (a harmful metabolite produced by fatty acid peroxidation) (Figure 1L). Since ferroptosis is a key pathological outcome of iron overload−induced oxidative stress, we further examined the ferroptosis markers ACSL4 and GPX4. However, no significant changes in these markers were observed in the kidneys of iron−overloaded mice (Supplementary Figure S1).

Similarly, the mitochondrial morphological changes observed in our model differ from the typical pathological features of ferroptotic mitochondria [39]: instead of exhibiting volume shrinkage, our mitochondria demonstrate swelling. Electron microscopy revealed mitochondrial swelling (cristae dissolution/fracture, matrix dissolution appearing flocculent or vacuolated) and increased cytoplasmic electron density in the FC group (Figure 1M). In vitro experiments revealed that normal TCMK−1 cells exhibited a higher mitochondrial membrane potential, with JC−1 forming aggregates that emitted red fluorescence. After 12 h of iron treatment, TCMK−1 cells stained with JC−1 showed monomer formation, emitting green fluorescence, indicating a lower mitochondrial membrane potential. The CCPC group was used as a positive control. The results showed that the mitochondrial membrane potential of TCMK−1 cells was significantly reduced after 12 h of iron treatment, and their function was severely impaired (Figure 1N). These data demonstrate that long−term iron intake triggers renal oxidative stress and tubular injury, ultimately leading to renal dysfunction.

### 3.2. Iron Overload Upregulates ALDH1a3 Expression in Renal Tubular Epithelial Cells

**Figure 2:**
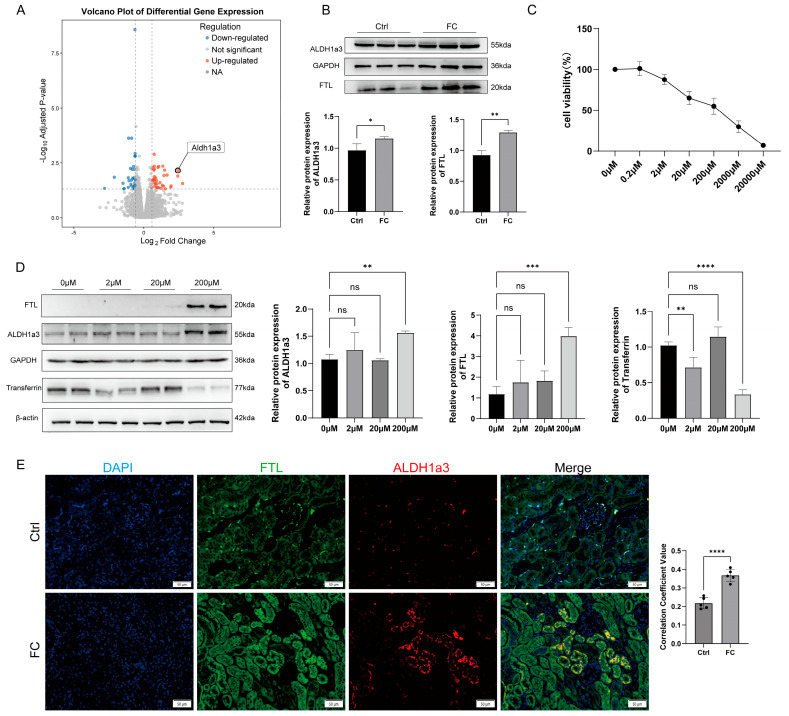
Iron overload upregulates ALDH1a3 expression in renal tubular epithelial cells. (**A**) Volcano plot of transcriptomic data (FC vs. Ctrl). (**B**) Western blot and quantification of ALDH1a3 and FTL in kidney tissue. (**C**) TCMK−1 cell viability after treatment with different concentrations of FAC (CCK−8). (**D**) Western blot and quantification of ALDH1a3, FTL, and Transferrin in TCMK−1 cells treated with FAC. (**E**) Co−localization immunofluorescence of FTL (green) and ALDH1a3 (red). All data are shown as mean ± standard deviation (SD). Statistical comparisons were performed using one−way ANOVA or t−tests. ns, not significant; * *p* < 0.05, ** *p* < 0.01, *** *p* < 0.001, **** *p* < 0.0001.

Transcriptomic analysis of kidneys from the FC group revealed a significant upregulation of ALDH1a3, a gene associated with retinal metabolism (Figure 2A). This increase was confirmed at the protein level (Figure 2B). Immunofluorescence co−staining for Ferritin Light Chain (FTL) and ALDH1a3 showed that ALDH1a3 expression increased concurrently with FTL expression in renal tubular epithelial cells (Figure 2E).

In vitro, TCMK−1 cells were treated with varying concentrations of ferric ammonium citrate (FAC) for 12 h (Figure 2C). At 200 μM FAC, transferrin expression decreased while FTL increased, indicating that cells had lost the capacity to transport excess iron and shifted towards storage, confirming intracellular iron overload. Consistent with in vivo findings, ALDH1a3 expression was upregulated in TCMK−1 cells under these conditions (Figure 2D). These results confirm that ALDH1a3 expression is induced during iron overload in renal tubular epithelial cells.

### 3.3. Overexpression of ALDH1a3 in TCMK−1 Can Resist Iron Overload−Induced Oxidative Stress and Mitochondrial Dysfunction

**Figure 3:**
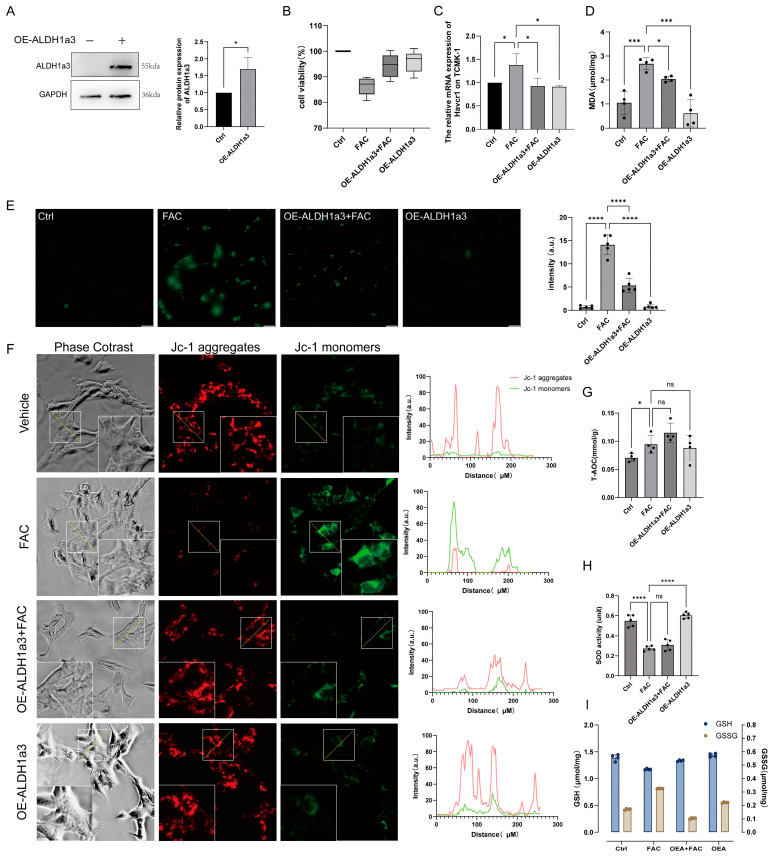
ALDH1a3 overexpression mitigates iron overload−induced oxidative stress and mitochondrial dysfunction. (**A**) Western blot and quantification of ALDH1a3 overexpression (OE− ALDH1a3). (**B**) Cell viability (CCK−8). (**C**) Havcr1 mRNA. (**D**) MDA content. (**E**) ROS detection (DCFH−DA). (**F**) JC−1 staining was used to assess the mitochondrial membrane potential, where red fluorescence indicates a higher membrane potential and green fluorescence indicates a lower membrane potential. The white box displays the enlarged view. (**G**) Total antioxidant capacity. (**H**) SOD activity. (**I**) GSH and GSSG content. All data are shown as mean ± standard deviation (SD). Statistical comparisons were performed using one−way ANOVA or t−tests. ns, not significant; * *p* < 0.05, *** *p* < 0.001, **** *p* < 0.0001.

To investigate the functional role of ALDH1a3, we overexpressed ALDH1a3 (OE−ALDH1a3) in TCMK−1 cells (Figure 3A). The OE−ALDH1a3 group exhibited improved cell viability after 12 h of iron intervention compared to Ctrl group (Figure 3B). Expression of the injury marker Havcr1 approached normal levels in OE−ALDH1a3 group (Figure 3C), and MDA levels were significantly lower than in the FAC group (Figure 3D). Furthermore, high levels of ROS induced by iron overload were suppressed in the OE−ALDH1a3 group (Figure 3E). JC−1 staining also revealed that overexpression of ALDH1a3 maintained an elevated mitochondrial membrane potential in TCMK−1 cells, thereby counteracting the decrease induced by FAC (Figure 3F). Compared to the group that received only FAC treatment, the FAC group with overexpressed ALDH1a3 exhibited higher levels of antioxidant capacity (Figure 3G). Interestingly, neither superoxide dismutase (SOD) activity nor catalase (CAT) activity exhibited significant changes, indicating that its antioxidant effect is independent of either the SOD pathway or the CAT pathway (Figure 3H, Supplementary Figure S2). However, the OE−ALDH1a3 group maintained high levels of reduced GSH and lower levels of oxidized GSH (GSSG) under prolonged iron stress (Figure 3I). This suggests that ALDH1a3 helps maintain a favorable GSH/GSSG ratio, indicative of a reduced cellular redox environment and enhanced antioxidant capacity.

In the detection of ferroptosis in the aforementioned iron−overloaded mouse kidneys, we did not observe significant changes in ferroptosis markers. However, under a single in vitro environmental condition, after subjecting TCMK−1 cells to 12 h of FAC treatment, we observed more severe cellular damage—not only alterations in oxidative stress levels but also the occurrence of ferroptosis. Our results demonstrate that in the FAC−treated group, the expression of ferroptosis marker ACSL4 is upregulated, while the expression of GPX4 is downregulated. Interestingly, after overexpressing ALDH1a3, we reversed FAC−induced ferroptosis and restored the expression levels of ACSL4 and GPX4 (Supplementary Figures S3).

### 3.4. Inhibiting ALDH1a3 in TCMK−1 Exacerbates Iron Overload−Induced Oxidative Stress and Mitochondrial Dysfunction

In the previous study, we preliminarily demonstrated the positive role of ALDH1a3 in resisting iron overload by overexpressing its function in TCMK−1 cells. To further validate the protective effect of ALDH1a3, we continued to use the ALDH1a3 inhibitor (ALDH1a3−IN3) to suppress ALDH1a3 function. First, we pretreated TCMK−1 cells with ALDH1a3−IN3 for 2 h, followed by combined treatment with inhibitors and FAC for 12 h.

**Figure 4:**
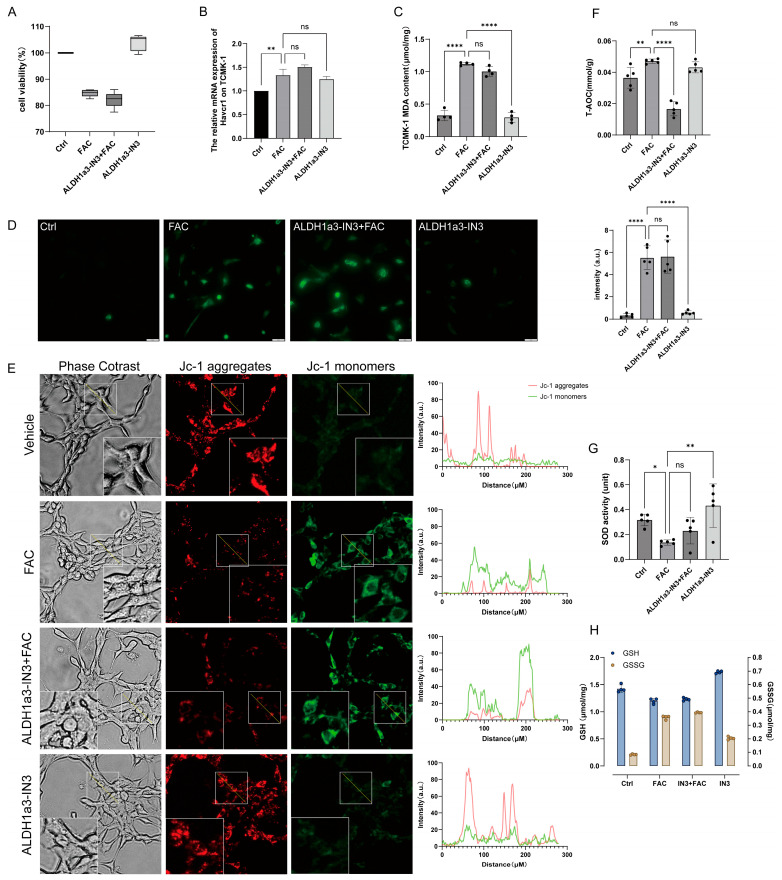
ALDH1a3 inhibition exacerbates iron overload−induced oxidative stress. (**A**) Cell viability with ALDH1a3 inhibitor (ALDH1a3−IN3). (**B**) Havcr1 mRNA. (**C**) MDA content. (**D**) ROS detection. (**E**) JC−1 staining was used to assess the mitochondrial membrane potential, where red fluorescence indicates a higher membrane potential and green fluorescence indicates a lower membrane potential. The white box displays the enlarged view. (**F**) Total antioxidant capacity. (**G**) SOD activity. (**H**) GSH and GSSG content. All data are shown as mean ± standard deviation (SD). Statistical comparisons were performed using one−way ANOVA or t−tests. ns, not significant; * *p* < 0.05, ** *p* < 0.01, **** *p* < 0.0001.

We found that the results generated after inhibitor intervention were opposite to those observed in the overexpression experiment. Cells treated with inhibitors exhibited lower survival rates (Figure 4A), higher levels of Havcr1 expression (Figure 4B), and persistently elevated malondialdehyde (MDA) levels (Figure 4C). The ALDH1a3−IN3 + FAC group showed no significant changes ROS levels or mitochondrial membrane potential (Figure 4D,E), but exhibited compensatory upregulation of superoxide dismutase (SOD) levels (Figure 4G). The CAT activity also showed a certain degree of upregulation, but this was not statistically significant (Supplementary Figure S4). The total antioxidant capacity of ALDH1a3−IN3 + FAC was further reduced (Figure 4F). Notably, while the reduced GSH levels were similar to those in the FAC group, the inhibitor group exhibited higher levels of oxidized GSSG (Figure 4H). Our loss−of−function experiments on ALDH1a3 demonstrated that inhibition of ALDH1a3 exacerbates iron−induced damage, which may be attributed to impaired antioxidant capacity.

In the cells of the ALDH1a3−IN3 + FAC group, we also detected ferroptosis markers. We observed that cells in the ALDH1a3−IN3 + FAC group remained in a ferroptosis state, with high ACSL4 expression and low GPX4 expression (Supplementary Figure S5). This finding is consistent with previous results and further demonstrates that ALDH1a3 contributes to reversing FAC−induced cell ferroptosis.

### 3.5. DP Alleviates Renal Iron Toxicity Stress and Is Accompanied by Regulation of ALDH1a3

**Figure 5:**
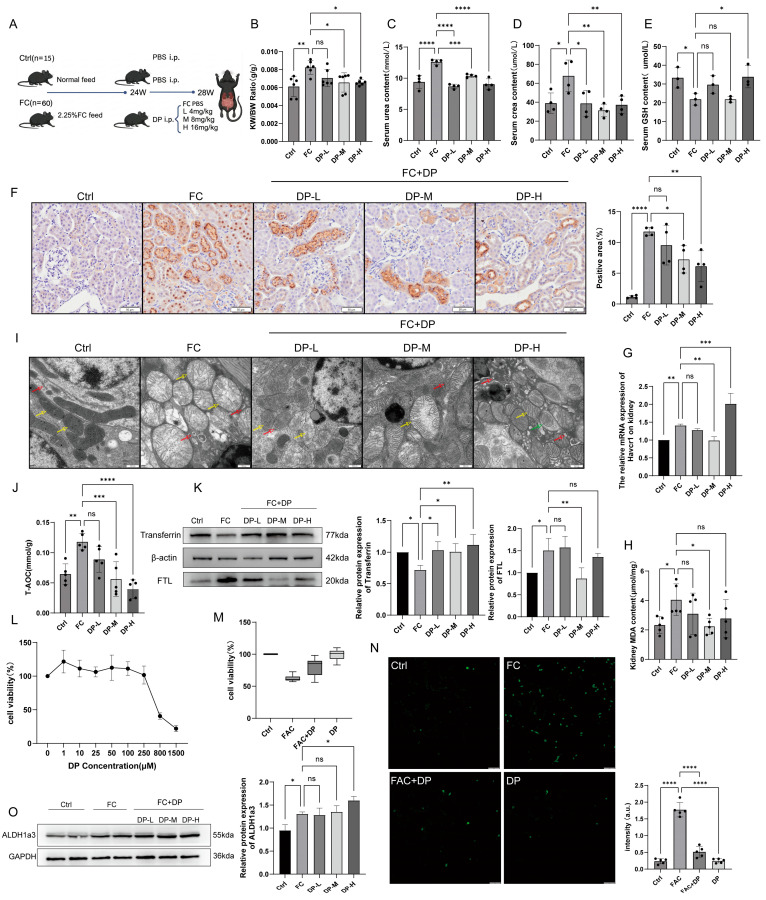
DP alleviates renal iron toxicity stress and is accompanied by regulation of ALDH1a3. (**A**) Experimental design for DP treatment (Low, Medium, High dose). (**B**) Kidney/body weight ratio. (**C**) Serum Urea levels. (**D**) Serum Crea levels (**E**) Serum GSH. (**F**) Prussian Blue staining and quantification of iron deposition. (**G**) Havcr1 mRNA. (**H**) Renal MDA. (**I**) Electron microscopy of mitochondria, the yellow arrows indicate mitochondria, the red arrows indicate the rough endoplasmic reticulum, and the green arrows indicate mitochondrial autophagy. (**J**) Total antioxidant capacity. (**K**) Western blot of Transferrin and FTL. (**L**) TCMK−1 viability with varying DP concentrations. (**M**) Rescue effect of 10 μM DP on FAC−treated cells. (**N**) ROS detection. (**O**) Western blot and quantification of ALDH1a3 in DP−treated kidneys. All data are shown as mean ± standard deviation (SD). Statistical comparisons were performed using one−way ANOVA or t−tests. ns, not significant; * *p* < 0.05, ** *p* < 0.01, *** *p* < 0.001, **** *p* < 0.0001.

Our previous research has shown that the acrolein scavenger dimercaprol provides neuroprotection in an animal model of Parkinson’s disease [40]. Iron overload leads to the production of lipid peroxides; therefore, it is worth investigating whether dimercaprol can intervene in iron overload by scavenging lipid peroxides. Following the intraperitoneal injection dosage in rats, we administered three doses of DP (low dose: 4 mg/kg; medium dose: 8 mg/kg; high dose: 16 mg/kg) to mice with an established renal iron overload model via intraperitoneal injection over a 4−week period (Figure 5A). Mice receiving different doses of DP were designated DP−L, DP−M, and DP−H, respectively.

Our results demonstrate that DP intervention at all doses restored organ indices and renal function (Figure 5B–D), recovered serum GSH levels (Figure 5E), and cleared renal iron deposits (Figure 5F). However, while the Medium dose (DP−M) resolved tubular injury, the High dose (DP−H) appeared to induce additional tubular damage (Figure 5G). DP−M was most effective in clearing renal MDA (Figure 5H). Morphologically, DP−L and DP−M groups showed a significant reduction in swollen mitochondria and restoration of basement membrane thickness. Conversely, the DP−H group exhibited increased cytoplasmic electron density and signs of organelle stress (ER dilation, lysosomal/mitochondrial autophagy) (Figure 5I). Endogenous antioxidant levels tended toward normal in the DP−M group (Figure 5J), and iron transport proteins (Transferrin and FTL) normalized (Figure 5K).

In vitro, 10μM DP treatment (Figure 5L) significantly improved survival (Figure 5M) and reduced ROS (Figure 5N) in iron−overloaded TCMK−1 cells. Importantly, ALDH1a3 expression increased in kidneys treated with DP (Figure 5O). This indicates that, in addition to its inherent iron chelation and detoxification of toxic aldehydes, the therapeutic effect of DP is also associated with upregulation of ALDH1a3 expression, a change that facilitates synergistic protection against iron−induced oxidative damage.

## 4. Discussion

In this study, we systematically characterized the significant renal tubular injury induced by ferric citrate (FC) mediated chronic iron overload. We identified oxidative stress, lipid peroxidation, and mitochondrial damage as core pathological features. Crucially, we have for the first time demonstrated that ALDH1a3 is upregulated as a key endogenous protective factor in the kidney under iron stress conditions. Through bidirectional experiments involving functional gain (overexpression) and functional loss (inhibitor intervention), we demonstrated that ALDH1a3 plays a critical role for resisting iron−induced oxidative damage in renal tubular epithelial cells and mitochondrial dysfunction. The ALDH1a3−IN−3 inhibitor exacerbates cellular damage and oxidative stress, further supporting that ALDH1a3 is a critical endogenous protective factor.

Although ALDH1a3 has been extensively studied for its role in retinoic acid synthesis and stem cell differentiation [41], our findings significantly expand its functional repertoire from a differentiation regulator to a protective factor against iron toxicity. The upregulation of ALDH1a3 in renal tubular epithelial cells facing iron overload suggests an environmentally responsive adaptation. ALDH1a3 may indirectly protect glutathione reductase activity by alleviating oxidative stress, thereby contributing to the maintenance of GSH pools. However, the specific mechanism by which ALDH1a3 affects the GSH/GSSG ratio remains unclear and requires further investigation. Our results also demonstrate that neither overexpression nor inhibition of ALDH1a3 significantly altered the activity of SOD and CAT, further supporting our conclusion that ALDH1a3 primarily functions through the GSH/GSSG cycle.

Furthermore, ALDH1a3 detoxifies lipid peroxidation−derived aldehydes (e.g., 4−HNE, MDA) that directly damage mitochondrial membrane proteins and cardiolipin [23]. By reducing aldehyde burden, ALDH1a3 preserves mitochondrial membrane integrity, prevents the opening of the mitochondrial permeability transition pore, and maintains electron transport chain function, thereby averting bioenergetic collapse [42].

Previous studies have demonstrated that ferroptosis is a key pathological outcome of iron overload−induced oxidative stress [43], yet typical features of ferroptosis—such as significant alterations in ferroptosis markers ACSL4 and GPX4 or characteristic mitochondrial shrinkage lesions—were not observed in our mouse kidneys with iron overload. In contrast to in vivo experiments, we observed alterations in ASCL4 and GPX4 levels in TCMK−1 cells treated with FAC. First, this may be related to the differences between the cellular survival environment and the complex environment within animals. The homogeneous nature of the cellular survival environment makes it more susceptible to ferroptosis under excessive iron induction compared to the in vivo animal environment. Secondly, due to limited conditions, the extracted renal tissues contained various types of renal cells rather than a single type of renal tubular epithelial cell, which is also one of the reasons for the observed differences. Importantly, in vitro experiments demonstrated that overexpression of ALDH1a3 inhibited FAC−induced ferroptosis. In future studies, we will conduct an in−depth investigation into the specific mechanisms by which ALDH1a3 inhibits ferroptosis.

Developing therapeutic strategies for iron overload−induced renal injury is a priority. Current interventions include iron chelators, antioxidants, and ferroptosis inhibitors [44,45,46,47,48]. However, monotherapy with chelators often presents challenges; high doses required for severe cases can increase the risk of adverse effects [49]. Dimercaprol (DP) offers potential advantages due to its ability to complex iron and potentially neutralize ROS. Our DP intervention model showed that while all doses cleared free iron, the therapeutic dose is critical. The low dose (4 mg/kg) and medium dose (8 mg/kg) were safe and effective, whereas the high dose (16 mg/kg) caused additional tubular injury. This highlights the critical importance of dose optimization when using chelating agents under iron overload conditions. Clinically, DP still requires strict monitoring to avoid nephrotoxicity.

In this study, we validated the protective role of ALDH1a3 against iron toxicity and observed its potential synergistic effects with DP therapy in vivo. Therefore, we hypothesize that DP not only directly chelates iron but also indirectly upregulates the expression of ALDH1a3, thereby enhancing the endogenous antioxidant system and contributing to improved therapeutic efficacy of DP. However, it remains to be further validated which signaling pathways DP activates to indirectly upregulate ALDH1a3. We propose a combined strategy: mild iron chelation (to reduce the toxic burden) coupled with pharmacological activation of ALDH1a3 (to enhance cellular tolerance). This approach could optimize treatment efficacy while minimizing chelator−associated side effects. ALDH1a3−mediated protection represents a promising complement to traditional iron chelation therapy. Although DP demonstrates promising therapeutic potential for renal iron overload, comparative data between DP and other compounds/chelators remain insufficiently investigated. Therefore, a thorough investigation into the advantages or disadvantages of DP compared to other compounds/chelators will enhance our understanding of its therapeutic role in iron overload disorders.

The present study has several limitations. First, hydrogen peroxide (H_2_O_2_) is a potent ROS inducer that directly accelerates the Fenton reaction and the conversion of Fe^2+^ to Fe^3+^ [50]. Future comparisons between FAC−treated and H_2_O_2_−treated groups will help distinguish iron−specific oxidative stress from general oxidative stress responses. Second, the SOD assay used in this study measured total SOD activity. Given that cytoplasmic SOD may dilute the positive signal, further evaluation of mitochondrial SOD (Mn−SOD) is crucial. Third, glutathione peroxidase (GPx) and glutathione reductase (GR) are key components of the GSH/GSSG cycle [51]. Measuring their levels would strengthen the evidence for ALDH1a3−mediated regulation of this pathway and is therefore a focus of future research. Fourth, we did not administer different doses of Dimercaprol (DP) to control animals, which precludes a precise assessment of DP’s intrinsic toxicity. Finally, future studies employing genetic knockout models are needed to elucidate the upstream signaling pathways that regulate ALDH1a3 upregulation in iron−overloaded kidneys, and metabolomic analyses should be conducted to identify the specific aldehyde substrates detoxified by ALDH1a3 in this context.

## 5. Conclusions

In summary, we have determined that the upregulation of ALDH1a3 expression in the context of renal iron overload serves as an endogenous protective factor against chronic iron overload−induced kidney injury. Its protective effect is closely related to reducing oxidative stress, increasing antioxidant substances, and maintaining mitochondrial stability. Our study not only deepens the understanding of iron nephrotoxicity but also demonstrates that ALDH1a3 is a promising potential therapeutic target for the treatment of iron overload−related kidney diseases.

## Data Availability

The data supporting this study are available from the corresponding authors upon reasonable request.

## Supplementary Materials

The following supporting information can be downloaded at: https://www.mdpi.com/article/10.3390/antiox15050577/s1. Figure S1: Western blot and quantification of ACSL4 and GPX4 expression in the kidneys of iron-overloaded mice. Figure S2: CAT activity after overexpression of ALDH1a3 in TCMK-1 cells. Figure S3: Western blot and quantification of ACSL4 and GPX4 expression after ALDH1a3 overexpression in TCMK-1 cells. Figure S4: CAT activity after ALDH1a3 inhibition in TCMK-1 cells. Figure S5: Western blot and quantification of ACSL4 and GPX4 expression after ALDH1a3 inhibition in TCMK-1 cells.

## Abbreviations

The following abbreviations are used in this manuscript:

ALDH1a3	Aldehyde dehydrogenase 1a3
CKD	Chronic kidney disease
Fe^3+^	Ferric iron
FC	Ferric citrate
MDA	Malondialdehyde
GSH	Glutathione
Havcr1	Hepatitis A virus cellular receptor 1
KIM−1	Kidney Injury Molecule−1
ROS	Reactive oxygen species
DP	Dimercaprol
ESRD	End−stage renal disease
SPF	Specific pathogen−free
Crea	Creatinine
qRT−PCR	Quantitative real−time PCR
SOD	Superoxide dismutase
FAC	Ferric Ammonium Citrate
GSSG	Oxidized glutathione
Nrf2	Nuclear factor erythroid 2−related factor 2
HIF−1α	Hypoxia−inducible factor 1−alpha
FTL	Ferritin Light Chain
MEM	Minimum Essential Medium
FBS	Fetal Bovine Serum
H&E	Hematoxylin and Eosin
