# Supplementary material for: ALDH1a3 Protects Against Iron Overload−Induced Oxidative Stress and Mitochondrial Impairment in Renal Tubular Epithelial Cells
Source: Antioxidants (Basel). 2026 May 2;15(5):577. doi: 10.3390/antiox15050577 (PMC13203110; doi:10.3390/antiox15050577)

Supplementary Figure. S1

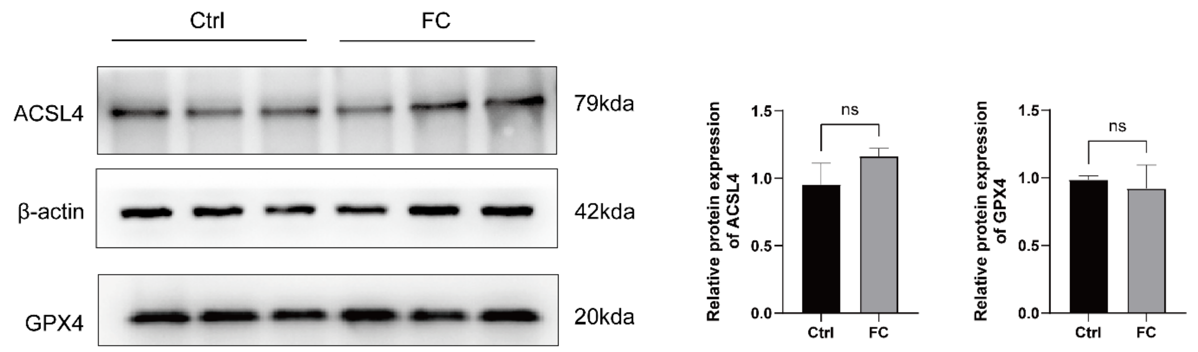

Supplementary Figure. S2

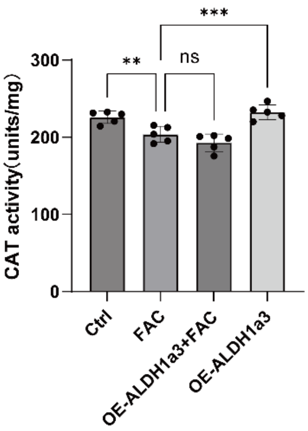

Supplementary Figure. S3

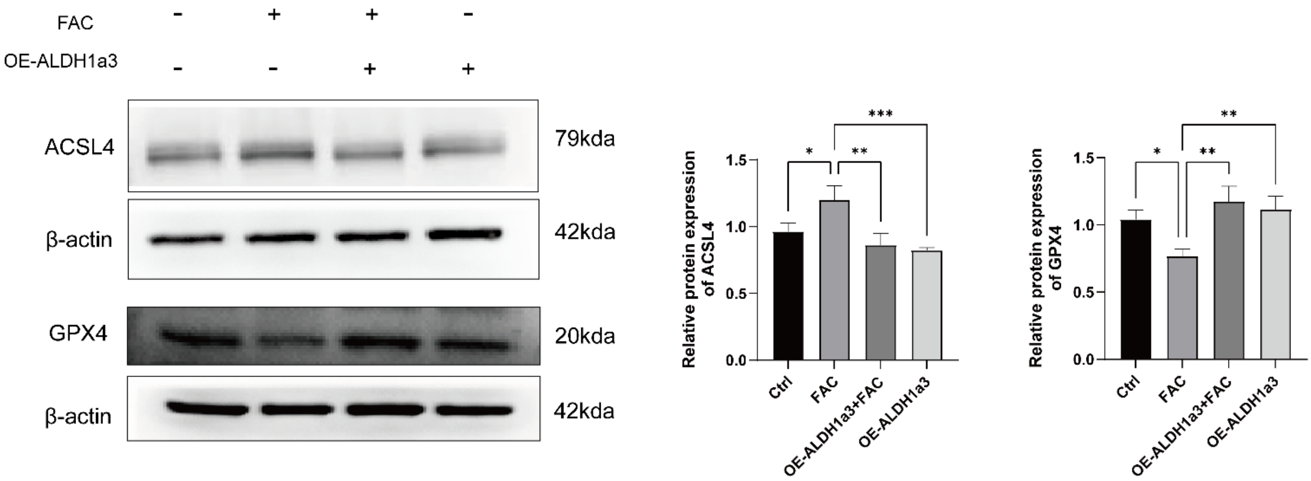

Supplementary Figure. S4

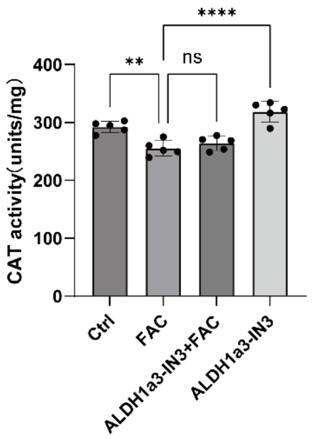

Supplementary Figure. S5

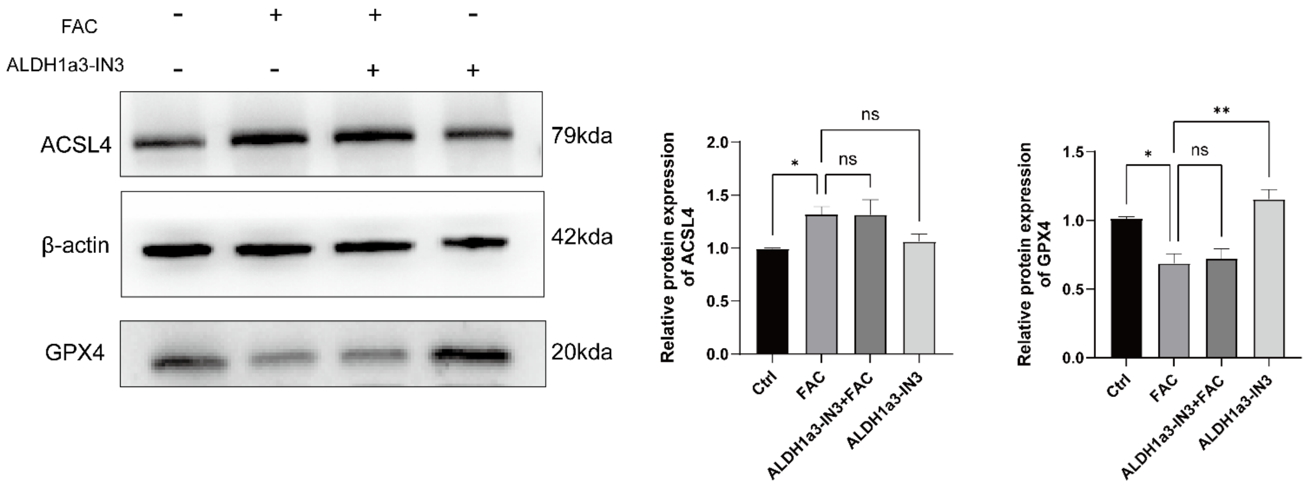

Supplement: Supplementary file 1 [file antioxidants-15-00577-s001.zip › antioxidants-4249455-supplementary.pdf]
